# Improving Patient Safety in Inpatient Psychiatry: A Closed-Loop Audit of Seclusion Review Quality

**DOI:** 10.1192/bjo.2026.11731

**Published:** 2026-06-30

**Authors:** Josephine Do, Emma Runswick, Samira Malik

**Affiliations:** Greater Manchester Mental Health NHS Foundation Trust, Manchester, United Kingdom

## Abstract

**Aims:**

Seclusion is highly restrictive and is a last resort to manage immediate risk of harm towards others. Therefore it requires high quality medical reviews and clear documentation to ensure patient safety.

**Aims:**

- To assess compliance with local trust standards for medical seclusion reviews in a inpatient mental health unit (Meadowbrook Unit, Salford, Manchester)
- Evaluate the impact of quality improvement measures through an re-audit.

Trust policy outlines eight core review criteria: assessment of physical and psychiatric health, medication and adverse effects, observations, risks to others and to self, and the ongoing need for seclusion. This is alongside two best-practice criteria addressing harms of seclusion and steps required to end it/patient awareness.

Quality improvement measures(local teaching/presentations, communication to line managers, and a poster of trust standards in clinical areas) were implemented after an initial audit in July 2024. The re-audit was conducted in March 2025.

**Methods:**

A retrospective audit of medical seclusion reviews was conducted using electronic clinical records within an inpatient mental health unit. Forty seclusion reviews from July–August 2024 were analysed against local trust seclusion review criteria. A re-audit of 56 seclusion reviews conducted between March and June 2025 was then undertaken to assess changes in practice.

**Results:**

The re-audit demonstrated higher quality seclusion reviews. No reviews met all eight criteria in 2024, but seven reviews did in 2025. The most common number of criteria met for each review increased from one criterion in 2024 (n=9) to four or five criteria in 2025 (n=12).

Documentation improved overall, including physical health (70% to 82%), psychiatric health (40% to 66%), and medication side effects (0% to 19%). Also, assessment of harms associated with ongoing seclusion improved (2% to 14%). Small declines were found in documentation of the decision to continue seclusion (97% to 91%) and steps to end seclusion or patient awareness (25% to 23%).

Limitations:

The 2024 audit had a smaller sample size (n=40) which was increased in the re-audit (n=56). The 2024 audit was also conducted shortly after a new doctors rotation. Patient sleep status(n=12), senior-led reviews (n=10), and undocumented reviews (n=3) in 2025 may have influenced findings however all reviews were included in the analysis.

**Conclusion:**

The re-audit demonstrated improvement in patient safety through higher quality of documented medical seclusion reviews. There was an improvement in assessment of physical and psychiatric health, medication, and risk assessment. Minor reductions were observed in documentation of decisions to continue seclusion and steps to end seclusion/patient awareness.

Further re-audit across other trust sites should occur with measures to address undocumented reviews and asleep reviews. There should be continued integration of seclusion standards into doctor induction programmes and regular local teaching.

This re-audit shows low-cost, targeted interventions can improve patient safety and seclusion review quality which is transferable to other inpatient mental health settings.

